# Rationale and protocol of a double-blind, randomized, placebo-controlled trial to test the efficacy, safety, and tolerability of dimethyl fumarate in Friedreich Ataxia (DMF-FA-201)

**DOI:** 10.3389/fnins.2023.1260977

**Published:** 2023-09-07

**Authors:** Chiara Pane, Alberto Maria Marra, Ludovica Aliberti, Mario Campanile, Federica Coscetta, Giulia Crisci, Roberta D'Assante, Angela Marsili, Giorgia Puorro, Andrea Salzano, Antonio Cittadini, Francesco Saccà

**Affiliations:** ^1^Department of Neurosciences and Reproductive and Odontostomatological Sciences, Federico II University, Naples, Italy; ^2^Department of Translational Medical Sciences, Federico II University, Naples, Italy; ^3^Cardiac Unit, AORN A Cardarelli, Naples, Italy

**Keywords:** clinical study design, Friedreich Ataxia, dimethyl fumarate, frataxin level, Nrf2 pathway, mitochondrial biogenesis, safety, clinical outcomes

## Abstract

**Introduction:**

Friedreich Ataxia (FRDA) is an autosomal recessive neurodegenerative disorder that causes gait and limb ataxia, dysarthria, and impaired vibratory sense, with cardiomyopathy being the predominant cause of death. There is no approved therapy, which results in the use of symptomatic treatments and the chronic support of physiotherapy. Dimethyl fumarate (DMF) is a fumaric acid ester used for the treatment of psoriasis and Multiple Sclerosis (MS). It induces Nrf2 *in vitro* and *in vivo*, and it increases frataxin in FRDA patient lymphoblasts, in mouse models, and in MS treated patients.

**Methods:**

The aim of our study is to investigate if DMF can increase the expression of the *FXN* gene and frataxin protein and ameliorate *in-vivo* detectable measures of mitochondrial dysfunction in FRDA. The study is composed of a screening visit and two sequential 12-week phases: a core phase and an extension phase. During the first phase (core), patients will be randomly assigned to either the DMF or a placebo group in a 1:1 ratio. During the first week, patients will receive a total daily dose of 240 mg of DMF or placebo; from the second week of treatment, the dose will be increased to two 120 mg tablets BID for a total daily dose of 480 mg. During the second phase (extension), all patients will be treated with DMF. EudraCT number 2021-006274-23.

**Endpoints:**

The primary endpoint will be a change in *FXN* gene expression level after 12 weeks of treatment. Secondary endpoints will be frataxin protein level, **c**ardiopulmonary exercise test outputs, echocardiographic measures, Nrf2 pathway and mitochondrial biogenesis gene expression, safety, clinical scales, and quality of life scales.

**Conclusions:**

This is the first study aimed at exploring the ability of DMF, an already available treatment for MS and psoriasis, to correct the biological deficits of FRDA and potentially improve mitochondrial respiration *in-vivo*.

## Introduction

### Background

Friedreich Ataxia (FRDA), an autosomal recessive neurodegenerative disorder, is the most common hereditary ataxia among persons of European ancestry (Harding, 1981). The disease is characterized by gait and limb ataxia, dysarthria, usually absent tendon reflexes, bilateral Babinski sign, and an impaired vibratory sense. Cardiomyopathy is the predominant cause of death (Filla et al., 2000).

The molecular defect is a trinucleotide GAA expansion in the first intron of the *FXN* gene (Campuzano et al., 1996) that encodes frataxin, a 210 amino acid mitochondrial protein. Compared to controls, FXN/mRNA is reduced to 20% in patients and to 53% in carriers (Saccà et al., 2011). In patients, frataxin protein is 36% of the level seen in controls and shows an inverse correlation with the size of the GAA repeat in the shorter allele (GAA1). Although the exact physiological function of frataxin is not known, it may have a role in iron–sulfur (Fe–S) cluster biogenesis, iron binding/storage, and scavenging against reactive oxygen species (ROS) (Bulteau et al., 2004).

All cells have an intrinsic protective mechanism from ROS that is controlled through the transcription factor Nrf2 that binds to the antioxidant response element (ARE) in the regulatory regions of target genes (Nguyen et al., 2003). Nrf2 is normally sequestered in the cytoplasm through interaction with Keap1, which results in constitutive ubiquitination and proteasomal degradation. Excessive ROS results in the modification of Keap1 such that Nrf2 is no longer constitutively degraded (Linker et al., 2011). A reduction in antioxidant defenses has been reported in fibroblasts from FRDA patients, highlighted by a deficiency in Nrf2 activation (Paupe et al., 2009), suggesting that FXN deficiency may cause lower Nrf2 activation and decreased mitochondrial antioxidant protection (Hayashi and Cortopassi, 2016).

Therapeutic strategies in FRDA include increasing frataxin protein and/or FXN/mRNA levels and replacing frataxin function (i.e., antioxidation or iron chelation). Antioxidants such as idebenone, a short chain quinone analog of CoQ10, are ineffective on the clinical course of the disease (Lynch et al., 2010). Erythropoietin (EPO) is a glycoprotein that acts as a main regulator for erythropoiesis. EPO increases frataxin levels in cultured human lymphocytes from FRDA patients. A phase IIb multicenter, randomized, placebo-controlled clinical trial showed no effect of Epoetin alfa on exercise capacity and on peak oxygen uptake (VO_2_max) at the cardiopulmonary exercise test (CPET) in FRDA patients (Saccà et al., 2016). Nicotinamide appears to be effective in increasing FXN/mRNA and frataxin protein levels when used at doses much higher than recommended, but several adverse events (AEs) occurred during the phase I trial of nicotinamide, and affected virtually all treated patients (nausea, lightheadedness, vomiting, and headache) (Libri et al., 2014). This will probably affect drug adherence and limit the long- term use of the drug. Considering that FRDA is a chronic condition, this may be a serious limitation to nicotinamide. Omaveloxolone is an activator of Nrf2 and restores mitochondrial function *ex vivo* in fibroblasts from people with FRDA, but there is no evidence that it can increase the levels of *FXN* gene expression and frataxin protein. Omaveloxolone significantly improved neurological function measured with the modified Friedreich's Ataxia Rating Scale (mFARS) compared to placebo and was generally safe and well tolerated (Lynch et al., 2021). The U.S. Food and Drug Administration (FDA) has recently approved Omaveloxolone as a treatment for FRDA.

Although several trials have been performed with FRDA patients, no effective treatment is available in Europe, and clinical practice consists of preventing the development of cardiomyopathy, and in the symptomatic management of the disease.

### Study rationale

A screening protocol for repurposed drugs that rescue frataxin-dependent defects in FRDA fibroblasts resulted in the identification of dimethyl fumarate (DMF) (Sahdeo et al., 2014), which dose-dependently increases frataxin in FRDA patient lymphoblasts.

DMF induces Nrf2 *in vitro* and *in vivo*, modifying cysteine residues in Keap1 (Linker et al., 2011). DMF also regulates HDAC expression and increases acetylation of H3.3 and H2A (Kalinin et al., 2013). Nrf2-binding sites exist in the *FXN* gene, as demonstrated by Chromatin Immuno- Precipitation (ChIP) experiments (Sahdeo et al., 2014), which represents one of DMF's modes of FXN induction.

Also, in the FRDA mouse models YG8 and KIKO with a dose range 3–10 mg/kg, DMF caused a dose-dependent increase in frataxin protein in the cerebellum and in multiple other tissues (Jasoliya et al., 2019). We recently found that DMF induces mitochondrial biogenesis and function in human fibroblasts and increases mitochondrial copy number, the mitochondrial biogenesis factor TFAM, 10 mitochondrial transcripts, and maximal mitochondrial O_2_ consumption rate (Jasoliya et al., 2019). NCF2 and PDLIM1 were also recently validated in human FRDA lymphoblast lines as markers of DMF treatment (Hayashi et al., 2017).

We performed an exploratory study to limit expenses and risks related to a straight phase II trial with DMF in patients with FRDA. We enrolled multiple sclerosis (MS) patients, for which a decision to start a therapy with DMF (*n* = 14) had already been taken as part of normal clinical practice, at the standard dose of 240 mg BID. We added to this study a parallel group of patients that started a treatment with fingolimod (*n* = 12), a recently approved oral treatment for MS, that served as a control group. We also enrolled twelve healthy controls. Baseline levels of FXN/mRNA in PBMCs were similar between controls and MS patients (*p* = 0.818). After 3 months of treatment, FXN/mRNA expression increased by 85.1% in the DMF group and 13.7% in the fingolimod group (relative increase in DMF +71.4%; *p* = 0.011). DMF also caused a 70% increase in mitochondrial copy number in PBMCs and an >100% increase in mitochondrial complex subunit expression of mt-ND6 (complex 1), mt-CYB (complex 3), mt-CO_2_ (complex 4) and mt-ATP6 (complex 5). The increase in these four transcripts could result in an *in-vivo* stimulation of mitochondrial biogenesis and improvement of oxidative phosphorylation, known to be impaired in FRDA patients (Hayashi et al., 2017).

Since MS patients showed comparable basal levels of FXN/mRNA to healthy controls, it is possible that DMF can increase FXN/ mRNA levels in healthy individuals as well as in patients with MS.

Based on the previous points, the aim of our study is to further investigate the role of DMF in FRDA and to demonstrate if DMF is able to correct the biological deficits of FRDA. The study will contribute to the understanding of the ability *in-vivo* of DMF, at currently approved doses for MS and psoriasis, to increase the expression of the *FXN* gene and to increase frataxin protein.

We propose a double-blind, placebo-controlled, randomized, phase II clinical trial to test the effect of DMF on FXN transcription in FRDA patients. The secondary objectives of the study will be the effect of DMF on frataxin protein, the nrf2 pathway, and mitochondrial biogenesis, safety and tolerability, and clinical aspects of the disease.

We hypothesize that DMF is superior to placebo in its ability to increase FXN/mRNA.

### The drug—dimethyl fumarate

DMF is a fumaric acid ester that has been used for the treatment of psoriasis (Rostami-Yazdi et al., 2010) due to its suppressive actions on pro-inflammatory T-cell activation and to shift T-cell polarity from Th1 to Th2 (Bovenschen et al., 2010). Recently, DMF received approval from the EMA for the treatment of psoriasis based on the positive results of the BRIDGE trial (Mrowietz et al., 2017). DMF has also been considered for the treatment of MS (Gold et al., 2012) and was approved for the treatment of relapsing remitting MS.

After oral intake, DMF is rapidly hydrolyzed by esterases to its active circulating metabolite monomethyl fumarate (MMF). DMF is currently administered due to its lower incidence of gastrointestinal side effects compared to MMF (Litjens et al., 2004). MMF's bioavailability is decreased by concurrent food ingestion, though it remains bioavailable. MMF is the most bioactive metabolite (Litjens et al., 2004) and typically reaches peak serum concentrations around 20 μM. MMF is eliminated mainly through breathing; only small amounts of intact MMF are excreted through urine or feces. There is no evidence for a cytochrome P450-dependent metabolism in the liver, thus few drug interactions would be expected. MMF's half-life *in vivo* is around 12 h. The safety profile of DMF (trade name: Skilarence) is well-known and adverse events of special interest that have been considered for Skilarence are (EMA/412737/2017, 2017) leukocytopenia and lymphopenia, flushing, gastrointestinal disorders, hepatic injury, malignancies, renal injuries and proteinuria, and serious and opportunistic infections. The reported incidence of leukocytopenia is 13.3%, and lymphopenia is 10.0%, with very few patients (<1%) experiencing severe enough lymphopenia (<0.5/L) to lead to discontinuation. Flushing has an incidence of up to 20.8% of patients, is more frequent during the first weeks of treatment, and tends to resolve during long-term administration of DMF. Gastrointestinal disorders were reported in 62.7% of patients receiving DMF. They usually resolved after the first few months of treatment. The proportion of patients with an increase in hepatic enzymes is 7.5% and all are usually mild/moderate intensity. There is no evidence that long-term treatment with DMF is associated with an increased risk of malignancies. The proportion of patients reporting renal injury/proteinuria is low (<3%). Several of these cases of renal toxicity occurred with doses of fumaric acid esters that are much higher than the dose recommended for DMF in psoriasis/MS. In all of these cases, the renal function returned to normal after stopping treatment. The number of infections during DMF treatment was very low (<5%). All infections resolved by the end of the studies or during the 2-month follow-up period. No relation was found between leukopenia/lymphopenia and infections.

## Methods and analysis

### Trial design and intervention

**Figure 1:**
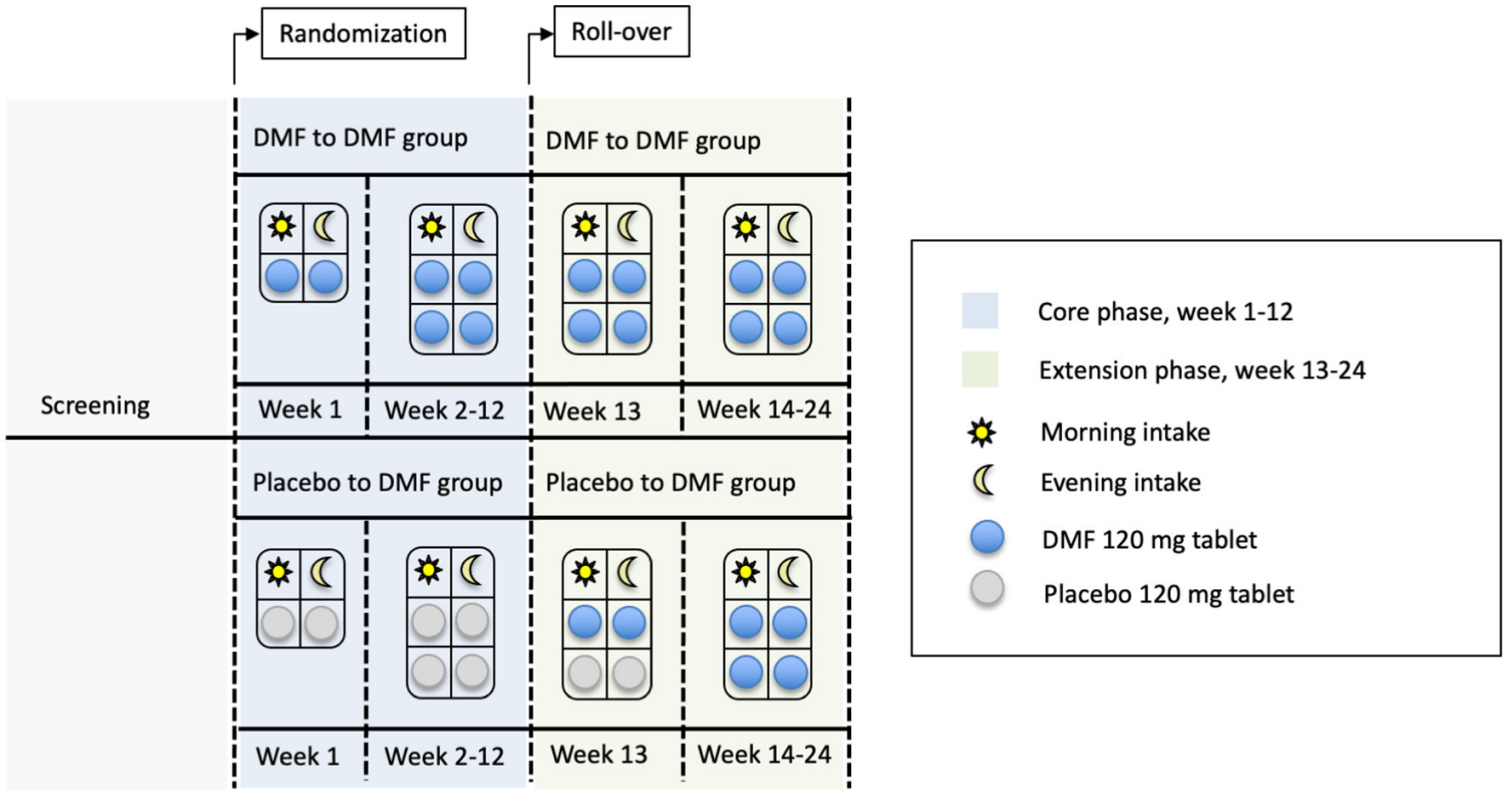
Drug disposition for the core and extension phase. DMF, dimethyl fumarate.

**Figure 2:**
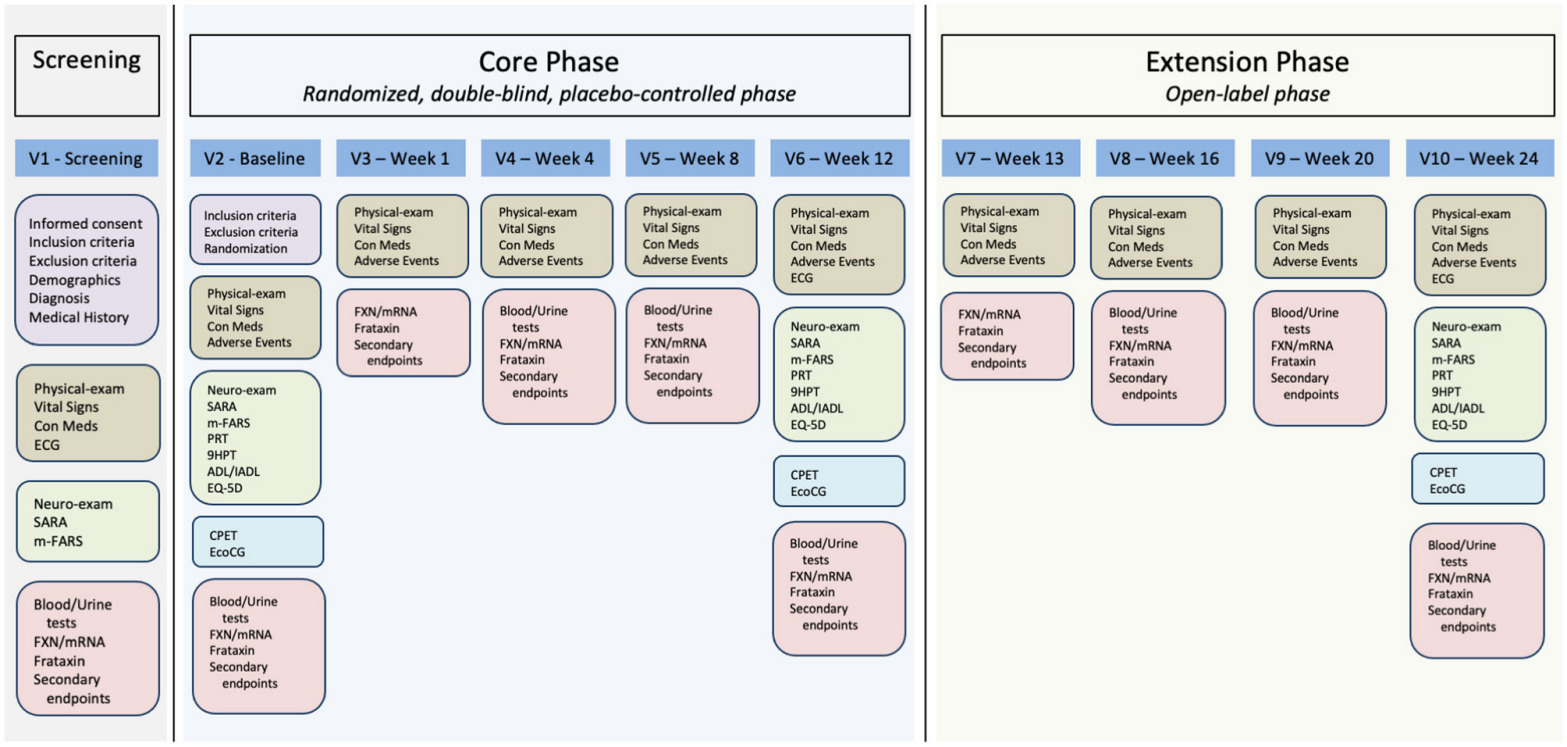
Study visit plan and procedures. V1, visit 1; V2, visit 2; V3, visit 3; V4, visit 4; V5, visit 5; V6, visit 6; V7, visit 7; V8, visit 8; V9, visit 9; V10, visit 10; ECG, electrocardiogram; Con Meds, concomitant medications; SARA, Scale for the Rating and Assessment of Ataxia; m-FARS, modified Friedreich Ataxia Rating Scale; FXN/mRNA, *FXN* gene mRNA measurement; PRT, PATA rate test; 9HPT, 9 hole pegboard test; ADL/IADL, activities of daily living/Instrumental activities of daily living; EQ-5D, European quality of life scale; CPET, cardiopulmonary exercise test; EcoCG, echocardiography.

This will be a single-center trial with only one center activated at the AOU Federico II University Hospital. The study is composed of a screening visit and two sequential 12-week phases: a core phase and an extension phase (Figures 1, 2). During the core phase, patients will be randomly assigned to either a DMF or placebo group. During the extension phase, all patients will be treated with DMF.

The use of placebo is based on several factors. On one side, there is a need for a more accurate evaluation on the biochemical effect on FXN/mRNA and frataxin that can show fluctuations over time. For this reason, an increase of FXN/mRNA in an open-label trial could be erroneously attributed to treatment. The second reason is that secondary endpoints (CPET, Clinical scales, etc.) can show a clear placebo effect once patients take part in a clinical trial. There is no known risk of being in the placebo group as there is no treatment available for FRDA.

The study will begin with a screening visit where patients will sign their informed consent (Figure 2). We will then assess inclusion and exclusion criteria (Table 1). Patients fulfilling all inclusion and none of the exclusion criteria will undergo endpoint measurement and will enter the core phase of the study.

**Table 1:** Inclusion and exclusion criteria.

**Inclusion criteria**
1. Molecular diagnosis of Friedreich Ataxia with a homozygous GAA expansion 2. Age ≥12 years 3. Body weight ≥30 Kg 4. Patients able to read and sign the informed consent form
**Exclusion criteria**
5. Treatment with DMF in the previous 12 months 6. Treatment with Idebenone, coenzyme Q10, or any other vitamin supplements in the previous 30 days 7. Patients in treatment with any other not allowed drug 8. Any Cardiac and/or Renal and/or Hepatic disease judged as clinically significant by the investigator (any abnormal and clinically non-significant cardiac disease associated with Friedreich Ataxia is not an exclusion criteria) 9. Any clinically significant ECG abnormalities that may interfere with the study 10. Any abnormal and clinically significant laboratory exams at screening visit that may interfere with the trial 11. Any acute disease that could interfere with the study, as judged by the investigator 12. Patient positive to the Human Immunodeficiency Virus (HIV) or Hepatitis B or C test 13. Patients with a positive history of neoplasia, with the only exception of a completely excided basal cell carcinoma 14. Positive history of alcohol or drug abuse in the past 2 years, except for medical use of cannabis 15. Hypersensitivity to DMF or any other component of the study drug 16. Patients not able to comply with the study 17. For female patients (Sexually inactive, hysterectomized, sterilized, or menopause patients are excluded from the following criteria): a) Pregnancy, or b) Breastfeeding, or c) Inadequate contraception

GAA, guanine-adenine-adenine triplet; DMF, dimethyl fumarate; ECG, electrocardiogram.

After entering the core phase, patients will be randomized to receive either DMF or placebo in a 1:1 ratio. Randomization will start with the retrieval of randomization lists that will be generated by the private CRO (FullCRO) involved in the study. Randomization will be in blocks of four and will be stratified based on GAA triplet repeat expansion (group I < 635; group II ≥635). For safety reasons, the treating physician will be able to break the code of a single patient and be informed of the allocation between active treatment and placebo. This will be followed by the immediate discontinuation of the patient.

For the first week of the core phase, patients randomized to the treatment group will receive one 120 mg tablet BID, for a total daily dose of 240 mg. Starting from the second week of treatment, the dose will be increased to two 120 mg tablets BID for a total daily dose of 480 mg (Figure 1). Rationale for the selected treatment dose is derived from the approved dose of DMF for Multiple Sclerosis and Psoriasis patients and the previously reported effect of DMF 240 mg BID in Multiple Sclerosis patients (Jasoliya et al., 2019). The placebo group will receive identical placebo tablets. Patients will refer to the study center for endpoint re-assessment and drug accountability procedures after 1, 4, 8, and 12 weeks. Patients completing the 12-week core phase will roll-over in a 12-week extension phase. Please see Figure 2.

During the extension phase, to maintain blindness to treatment allocation, at week 13, patients receiving DMF in the core phase will continue with DMF 480 md/day in the form of two 120 mg DMF tablets BID. Patients receiving placebo in the core phase will receive one tablet of DMF 120 mg BID and one placebo tablet BID. From week 14 to 24, all patients will receive two 120 mg DMF tablets BID. Patients will again be visited after 1, 4, and 12 weeks after entering in the extension phase. A detailed description of the study activities is shown in Table 2.

**Table 2:** Visit schedule.

**Phase**		**Core Phase**					**Extension phase**				**EOS**
**Visit**	**1**	**2**	**3**	**4**	**5**	**6**	**7**	**8**	**9**	**10**	
**Activities and evaluations**	**Screening (**−**1 to** −**2 weeks)**	**Baseline**	**Week 1–day 7 (**±**1 day)**	**Week 4–day 28 (**±**4 days)**	**Week 8–day 56 (**±**1 week)**	**Week 12–day 84 (**±**1 week)**	**Week 13–day 91 (**±**1 day)**	**Week 16–day 112 (**±**4 days)**	**Week 20–day 140 (**±**1 week)**	**Week 24–day 168 (**±**1 week)**	**Study interruption**
Informed consent	X										
Inclusion/exclusion criteria	X	X									
Demographics	X										
Diagnosis	X										
Past and actual medical history	X										
Randomization		X									
Physical examination and vital signs	X	X	X	X	X	X	X	X	X	X	X
Concomitant therapies	X	X	X	X	X	X	X	X	X	X	X
ECG	X					X				X	X
Neurological examination	X	X				X				X	X
SARA	X	X				X				X	X
m-FARS	X	X				X				X	X
PATA rate Test		X				X				X	X
9HPT		X				X				X	X
CPET		X				X				X	X
ECOCG		X				X				X	X
EQ-5D		X				X				X	X
ADL/IADL		X				X				X	X
Drug administration/accountability		X	X	X	X	X	X	X	X	X	X
Adverse events		X	X	X	X	X	X	X	X	X	X
Routine biochemistry	X	X	X	X	X	X	X	X	X	X	X
Hematology	X	X	X	X	X	X	X	X	X	X	X
Urine examination	X	X	X	X	X	X	X	X	X	X	X
Blood sampling for protein/RNA	X	X	X	X	X	X	X	X	X	X	X
Pregnancy test	X			X	X	X		X	X	X	X

ECG, electrocardiogram; SARA, Scale for the Rating and Assessment of Ataxia; m-FARS, modified Friedreich Ataxia Rating Scale; 9HPT, 9 hole pegboard test; ADL/IADL, activities of daily living/instrumental activities of daily living; EQ-5D, European quality of life scale; CPET, cardiopulmonary exercise test; EcoCG, echocardiography.

### Study population

The trial will enroll patients from our outpatient clinic of hereditary ataxias at the AOU Federico II University. Our outpatient clinic currently follows 120 patients with molecular diagnosis of FRDA with regular bi-annual visits. An additional number of patients attend the center on a less regular basis. Patients' clinical and molecular data are stored in an electronic database that will be used for pre-screening purposes. We will enroll patients based on prescreening procedures, i.e., already known patients, through enrollment calls via the Italian association for Ataxia (AISA).

During our last phase II trial with EPO, we managed to recruit 46 patients in a 24-week interval (Saccà et al., 2016). For that study, inclusion/exclusion criteria were more stringent as patients were required to be able to perform a cardiopulmonary exercise test with an arm ergometer. We were able to include patients with a SARA score up to 32 and have them perform the CPET. Based on the inclusion/exclusion criteria of this trial, we will be able to complete enrollment during a 24-week interval.

Based on inclusion criteria, and in a similar way to the previous trial (Saccà et al., 2016), we will include patients with an age equal to or higher than 12 years. The reason for dosing minors is that FRDA does affect teenagers and children. This group is considered as a whole; exclusion of minors would lead to biased results and exclude patients who would benefit more from an innovative treatment. In addition, the use of DMF in minors does not require any additional test, nor did it raise any additional side effects as compared to adults. In the present study, it will be up to the physician's discretion to exclude patients not able to perform CPET from the trial.

The study will have specific withdrawal criteria linked to the safety of DMF treatment that can be found in Table 1. This will be an abnormal laboratory result showing abnormalities in any of the following: lymphocyte count (<0.5/L), neutrophils (<0.5/L), AST (>5 × UNL), or ALT (>5 × UNL). In addition to these specific criteria, the treating physician will be free to exclude patients with any adverse event or laboratory results that he/she considers life threatening or that could interfere with the study. In the case of withdrawal, patients will be monitored with laboratory exams and clinical visits as needed until resolution of the abnormality. Throughout the trial, and starting from a pre-specified time before screening in parenthesis, patients will not be allowed on the following drugs: DMF (180 days), Idebenone and other coenzyme Q10 analogs (30 days), Nicotinamide (90 days), Nicotinic acid (90 days), Valproic acid (30 days), Interferon-gamma (90 days), Erythropoietin (180 days), Etravirine (180 days), or any experimental drugs (180 days). The rationale for prohibited medications is the demonstrated *in-vitro* or *in-vivo* ability to increase FXN/mRNA or frataxin levels.

### Endpoints

#### Primary endpoints

The primary objective of the study is to evaluate the effect of DMF compared to placebo on *FXN* gene expression level considering the change from baseline to 12 weeks (core phase of the DMF-FA-201 study).

#### Secondary endpoints

The secondary clinical objectives are to examine changes on the cardiopulmonary exercise outputs (VO_2_max, anaerobic threshold, and peak workload) and echocardiographic measures; and to evaluate differences in the Scale for the Rating and assessment of Ataxia (SARA), modified Friedreich Ataxia Rating Scale (m-FARS), 9-hole pegboard test (9HPT), EQ-5D, Activities of Daily Living (ADL)/Instrumental Activities of Daily Living Scale (IADL), and number and distribution of serious and non-serious adverse events between DMF and placebo.

The secondary laboratory objectives are to determine the effect of DMF on *FXN* and frataxin protein considering the change from baseline to week 12 and week 24 and at week 1 of the core phase, and at week 1 of the extension phase for those patients transitioning from placebo to DMF, compared to other time-points obtained after a DMF dose of 240 mg BID. Other secondary endpoints explore the effect of DMF on Nrf2 pathway genes (*NFE2L2, NQO1, HMOX1, PDLIM1*, and *NCF2*), on mitochondrial biogenesis genes (mt-ND6, mtCYB, mt-CO_2_, and mt-ATP6), and on mtDNA/nDNA.

### Clinical measures

An independent rater, blinded to laboratory results and side effects, will measure clinical endpoints (SARA and 9HPT). This will be necessary in order to assure that clinical measures will not be influenced by the treating physician in the case they accidentally recognize treatment allocation, based on side effects or laboratory results. EQ-5D and ADL/IADL will be self-rated. FXN/mRNA and frataxin protein are recognized endpoints for phase II clinical trials in FRDA patients (Libri et al., 2014). The investigation of the Nrf2 pathway and mitochondrial biogenesis genes are the logical consequence of DMF's mechanism of action and should be closely monitored during a clinical trial.

#### The scale for the rating and assessment of ataxias and the modified Friedreich Ataxia Rating Scale

The SARA scale consists of eight items (gait, stance, sitting, speech disturbance, finger chase, nose-finger test, fast alternating hand movements, and heel-shin slide). The scale can score from 0 to 40, with 40 being the worst condition. The scale was validated in FRDA and is the current European standard for the measurement of ataxic symptoms over time (Bürk et al., 2009). It has been the standard for previous clinical trials (Saccà et al., 2016).

The m-FARS includes a bulbar, upper and lower limb coordination, and upright stability tests. The scale ranges from 0 to 85. It is a modified version of the original FARS and has been approved by the FDA as a clinical outcome for registration trials.

#### The index of independence in activities of daily living and the instrumental activities of daily living scale

ADL is an appropriate measure to monitor changes in daily self-care activities in FRDA patients (Reetz et al., 2016; Pane et al., 2018). It is an instrument to assess functional status as a measurement of the patient's ability to perform activities of daily living independently. The Index ranks adequacy of performance in the six functions of bathing, dressing, toileting, transferring, continence, and feeding. Patients are scored yes/no for independence in each of the six functions. A score of 6 indicates full function, 4 indicates moderate impairment, and 2 or less indicates severe functional impairment.

IADL is an instrument to assess independent living skills. These skills are considered more complex than the basic activities of daily living as measured by ADL. The instrument is most useful for identifying how a person is functioning at the present time, and to identify improvement or deterioration over time. Patients are scored according to their highest level of functioning in that category. A summary score ranges from 0 (low function, dependent) to 8 (high function, independent) for women, and 0 through 5 for men.

#### 9-hole pegboard test

The 9-HPT is a brief, standardized, quantitative test of upper extremity function. Both the dominant and non-dominant hands are tested twice. On a start command when a stopwatch is started, the patient picks up the nine pegs one at a time as quickly as possible, puts them in the nine holes, and, once they are in the holes, removes them again as quickly as possible one at a time, replacing them into the shallow container. The total time to complete the task is recorded. Two consecutive trials with the dominant hand are immediately followed by two consecutive trials with the non-dominant hand.

#### PATA rate test

The PRT allows for a quick screening test to assess dysarthria. Probands will be invited to repeat the syllables “PA-TA” as quickly as possible during a 10-s interval. The test will be immediately repeated for a second time. Both trials are then averaged for final score determination (Reetz et al., 2021).

#### The cardiopulmonary exercise test

CPET will be performed using an upper limb cycle ergometer (Ergoselect 400, Ergoline GmbH, Blitz, Germany). A ramp protocol of 5 W/min will be used and continued until limiting symptoms, chest pain, signs of ischemia, or arrhythmias develop or other indications for exercise termination appear. Subjects will be instructed to keep pedaling at a constant rate (50–60 rpm) during the test. Subjects will be advised that they will be free to stop whenever they wish but will be encouraged to continue for as long as possible. Respiratory gas exchange measurements will be obtained breath by breath by a commercially available system (Vmax 29C; Sensormedics, Yorba Linda, CA, USA). Peak oxygen uptake (VO_2_max) and respiratory exchange ratio (RER) will be recorded at the mean value of VO_2_ during the last 20 s of the test. The ventilatory anaerobic threshold will be detected by the use of the V-slope method. The ventilation per minute (VE) vs. carbon dioxide production (VCO_2_) relationship (ventilatory efficiency) will be measured by plotting ventilation against VCO_2_ obtained every 10 s of exercise (VE/VCO_2_ slope). The VE/VCO_2_ slope will be calculated as a linear regression function, excluding the non-linear part of the relationship after onset of acidotic drive to ventilation. Peak exercise oxygen pulse will be calculated by dividing derived VO_2_max by the maximum heart rate (HR) during exercise and will be expressed in milliliters per beat. HR will be recorded by ECG at rest, at the anaerobic threshold (AT), and at peak exercise (Guazzi et al., 2018; Pritchard et al., 2021).

There will be a body weight cut-off at 30 Kg based on the need for an appropriate estimation of VO_2_ calculation in children that cannot be reliable for a lower body weight.

#### Echocardiography

An ultrasound system equipped with a 2.5 MHz multifrequency transducer (Aplio, Toshiba, Japan) will be used for complete M-mode, two-dimensional, Doppler, and Tissue Doppler Imaging (TDI) echocardiographic analyses. M-mode and two-dimensional recordings will be made with the patients in the lateral recumbent position. Measures of LV end-diastolic volume (EDV) and end-systolic volume (ESV) will be measured by the modified Simpson's rule. Accordingly, ejection fraction (EF) will be calculated as follows: EF = (EDV – ESV)/ESV^*^100. LV mass will be calculated according to the America Society of Echocardiography-recommended formula: LV mass = 0.8^*^{1.04[(LVIDd + PWTd + SWTd)^3^ – (LVIDd)^3^]} + 0.6 g where PWTd and SWTd are posterior wall thickness at end diastole and septal wall thickness at end diastole, respectively. The following parameters of diastolic function will be measured as the mean of three to five consecutive beats: diastolic transmitral peak velocities, E/A ratio, Isovolumic Relaxation Time (IRT), mitral deceleration time, and pulmonary vein velocities. Quantitative diastolic data will be derived from TDI analysis as elsewhere described.

#### Quantitative real time PCR

Total mRNA will be extracted from PBMCs using TRIzol^®^ reagent (Thermofisher) following manufacturer's instructions. Using the one-step High Capacity RNA-to-cDNA Master Mix (Thermofisher, Carlsbad, CA, USA), 1 μg mRNA will be reversely transcribed following manufacturer's instructions in a total volume of 20 μl. About 2 μl of cDNA will be amplified using the TaqMan^®^ Gene Expression Master Mix and TaqMan^®^ Gene Expression Assay for frataxin (Applied biosystems, catalog no. Hs00175940_m1) in a StepOne real-time PCR. We will test 10 reference genes in order to choose the most stable in our experimental conditions using normfinder software. Relative expression will be calculated with the efficiency-calibrated model. The entire procedure will follow the Minimum Information for Publication of Quantitative Real-Time PCR Experiments (MIQE) guidelines.

#### Frataxin measurement

Peripheral Blood Mononuclear Cells will be extracted from 15 ml of EDTA anticoagulated whole blood and stored until analysis. PBMCs will be lysed with an extraction buffer (Abcam n.AB193970-50, Cambridge, UK), and total protein will be measured using the bicinchoninic acid assay. Each sample will be assayed in duplicate using a commercially available ELISA kit (Frataxin profiling ELISA kit, n.AB110173, Abcam, Cambridge, UK), and will be normalized with a standard curve using full length human frataxin (n.AB110353, Abcam, Cambridge, UK). This method will be used to measure frataxin at baseline and at weeks 4, 12, and 24 of the study.

### Electronic case report form

All variables will be stored in the electronic case report form (eCRF) of the study that will be specifically designed for the present study using Filemaker 19.5 software. It will be accessible through the web with a common browser. It will be hosted on a server based at the coordinating center with RAID type data protection. The eCRF will follow all indications of the Italian drug agency (AIFA) for non-profit clinical trials, including audit-log trail, data protection and backup, sheet freezing, and data unlock for deviations.

### Sample size calculation

Sample size calculation was performed based on our preliminary results of DMF treatment in patients with Multiple Sclerosis and is based on the estimated increase in FXN expression with the following assumptions derived from our preliminary data (Jasoliya et al., 2019). We previously found a significant +85% increase in the group treated with DMF compared to no increase in the control group (time^*^treatment interaction *p* = 0.02), which resulted in a partial eta square of 0.198. The sample size in this study was 14 patients treated with DMF and 13 with reference control treatment. The partial eta square of 0.198 results in an effect size of 0.497. Estimating the effect size based on preliminary data on MS patients is an underestimation of the potential effect of DMF in FRDA patients. Indeed, DMF can overexpress FXN through two mechanisms. One is the stimulation of nrf2 and the increase in transcription of several genes, including antioxidant genes and frataxin. This mechanism is clearly involved in MS patients treated with DMF. The second mechanism is the ability of DMF to induce transcription initiation and reduce transcriptional pausing in mutant *FXN* gene. This mechanism is unique to FRDA patients as it requires a mutated gene to be effective. In support of this, experiments in patients' lymphoblasts treated with DMF have found up to a +260% increase relative to baseline (Lynch et al., 2010), compared to our finding of +85% increase in MS patients treated with DMF. Based on this data, we calculated the appropriate sample size for this study using G^*^Power version 3.1.9.6. We used an ANOVA for repeated measures, within-between interaction, as the appropriate test. Effect size was set at 0.497, alpha error at 0.05, power at 0.8, number of groups 2, number of measurements 2, and non-sphericity correction 1. This results in a total population of 36 patients (18 treated + 18 placebo). We will prudently include 40 patients in total, allowing us to compensate for a lost at follow-up of up to 10% of randomized patients.

### Statistical analysis

All data will be preliminary tested for normality using the Kolmogorov–Smirnov test. Since quantitative real time PCR (qPCR) data are frequently not normal, a normalization attempt, using ln or log transformation, will be performed. qPCR data will be calculated as relative increases imputing a baseline expression level of 1.0. Main statistical analysis of both FXN expression and frataxin protein level will be conducted with a Generalized Linear Model (GLM) for repeated measures. GLM incorporates a two-way ANOVA for repeated measures and can accommodate a multivariate analysis of factors and covariates. Treatment with DMF or placebo will be considered as a factor, and baseline levels of frataxin will be considered as a covariate. FXN baseline expression will not be used in the model as it is already integrated using relative expression with common baseline. All other available data (sex, age, disease duration, GAA1, and treatment specific findings, etc.) will be preliminarily tested one by one as covariates. In case of resulting *p*-values < 0.1, they will be integrated in the final multivariate model with treatment as a factor. In case of a significant Maulchy's sphericity test, we will use the Greenhouse-Geisser correction. We will use a similar analysis approach for remaining secondary endpoints. This will include different time-points from FXN and frataxin, and other qPCR analysis, SARA, 9HPT, PRT, ADL/IADL, and EQ-5DVAS. The main analysis will be conducted with results from the core phase of the trial (20 patients treated with DMF and 20 with placebo) and will be used to draw conclusions on the efficacy of DMF. A secondary analysis will be conducted adding to the model patients from the extension phase that switched from placebo to DMF (total of 40 DMF vs. 20 placebo). This analysis will be considered as descriptive and not used to drive efficacy conclusions. For adverse events (AEs), we will perform a descriptive analysis of AEs including their distribution in DMF and placebo treated patients, AEs duration, severity, and connection to the study drug. To test if treatment with DMF increases the likelihood of having an AE, we will use a Generalized Linear Model with Poisson distribution and treatment as a factor. To test if a single AE is more frequently linked to DMF treatment, we will use the exact test of Goodness of fit, assuming an equal proportion of adverse events in the treatment and placebo group. This will be possible only for adverse events occurring at least once in both groups. For baseline variables a descriptive analysis will be performed, and difference between DMF and placebo patients will be tested with an unpaired *t*-test and chi-square test, depending on the variable. In case of non-normal distribution, a Mann–Whitney test will be used in place of a *t*-test. A two-sided significance level of 5% will be required to reject the null hypothesis that the outcome measures are the same before and after treatment, and for all other comparisons. The Bonferroni correction for multiple analyses will be used. Considering the primary endpoint and the phase of the trial, the analysis will be per protocol. Data will be analyzed using the SPSS 23 for MAC (IBM, Chicago, USA).

### Good clinical practice

Physicians will perform the study in accordance with ICH Good Clinical Practice and Good Clinical Practice for Trials on Medicinal Products in the European Community (ISBN 92-825-9563-3). The Investigator is required to ensure his/her compliance to procedures required by the protocol. The Investigator agrees to provide all information requested in Case Report Form in an accurate manner. The Investigator is required by Italian Health Authorities to ensure the proper conduct of the study as regards ethics, protocol adherence, integrity, and validity of the data recorded on the case report forms. Specific hazards for this study are the respect of inclusion and exclusion criteria. It is critical that all patients are correctly diagnosed with a molecular test and that this is available as a documentation of the study. Not allowed medications are another critical point as they could interfere with the study drug. This will be accurately checked by the study monitor before patients receive their first treatment. It is also critical that patients comply with the drug dose regimen, and this will be checked through compliance pill counts, house diaries, and drug accountability logs. Monitors will be required to check all of these procedures at each visit. Data generated during the study, and that will be collected in the eCRF, will be laboratory results, clinical scales, self-reported scales, concomitant treatment, and adverse events. All will be monitored at each visit for 100% coverage of all imputed data. A total of six monitoring visits will be performed during the study.

Compliance with the study protocol and risk minimization will be achieved through training of all site personnel at the beginning of the study and with periodical (every 3 months) meetings with update on enrollment, side effects, monitoring findings, DMF new SUSARs, and adherence to the programmed schedule. Meeting outcomes will be shared with the study personnel and with the study monitor.

## Discussion

The present trial is of extreme interest for healthcare providers as no approved therapy for FRDA exists in Europe. This results in the use of symptomatic therapy for the treatment of cardiac and orthopedic complications, diabetes, depression, and urinary disturbances, and in the chronic support through physiotherapy.

Recently, Omaveloxolone has been approved in the US by the FDA to treat FRDA patients. Despite a pending approval from EMA, there is still a need for multiple therapeutic choices for the disease, for improved disease modifying effect, and for more robust effects on the primary molecular defect (i.e., low frataxin expression).

Current therapies cannot halt disease progression, which occurs over time. Onset occurs usually when patients are in their teenage years and causes patients to be wheelchair-bound ~10 years after diagnosis. The result is that the social costs for such disability are both direct, for the costs of physiotherapy and symptomatic drug management, but also indirect for their loss in productivity. The availability of an effective treatment able to halt or slow disease progression may be of immense value as it may help reduce both healthcare and social costs of the disease.

## Ethics statement

The studies involving humans were approved by the Clinical Trial Information System (CTIS) with the ID number 3873. The studies were conducted in accordance with the local legislation and institutional requirements. The participants provided their written informed consent to participate in this study.

## Author contributions

CP: Writing—original draft, Writing—review and editing. AMM: Writing—review and editing. LA: Writing—review and editing. MC: Writing—review and editing. FC: Writing—review and editing. GC: Writing—review and editing. RD'A: Writing—review and editing. AM: Writing—review and editing. GP: Writing—review and editing. AS: Writing—review and editing. AC: Writing—review and editing. FS: Conceptualization, Data curation, Funding acquisition, Investigation, Methodology, Project administration, Supervision, Visualization, Writing—original draft, Writing—review and editing.

## References

[B1] Bovenschen H. J. Langewouters A. M. van de Kerkhof P. C. (2010). Dimethylfumarate for psoriasis. Am. J. Clin. Dermatol. 11, 343–350. 10.2165/11533240-000000000-00000 20553063

[B2] Bulteau A.-L. O'Neill H. A. Kennedy M. C. Ikeda-Saito M. Isaya G. Szweda L. I. . (2004). Frataxin acts as an iron chaperone protein to modulate mitochondrial aconitase activity. Science 305, 242–245. 10.1126/science.1098991 15247478

[B3] Bürk K. Mälzig U. Wolf S. Heck S. Dimitriadis K. Schmitz-Hübsch T. . (2009). Comparison of three clinical rating scales in Friedreich ataxia (FRDA). Mov. Disord. 24, 1779–1784. 10.1002/mds.22660 19562766

[B4] Campuzano V. Montermini L. Moltò M. D. Pianese L. Cossée M. Cavalcanti F. . (1996). Friedreich's ataxia: autosomal recessive disease caused by an intronic GAA triplet repeat expansion. Science 271, 1423–1427. 10.1126/science.271.5254.1423 8596916

[B5] EMA/412737/2017 (2017). Committee for Medicinal Products for Human Use (CHMP). Available online at: https://www.ema.europa.eu/en/committees/committee-medicinal-products-human-use-chmp (accessed April 21, 2017).

[B6] Filla A. De Michele G. Coppola G. Federico A. Vita G. Toscano A. . (2000). Accuracy of clinical diagnostic criteria for Friedreich's ataxia. Mov. Disord. 15, 1255–1258. 10.1002/1531-8257(200011)15:6&lt;1255::AID-MDS1031&gt;3.0.CO;2-C 11104216

[B7] Gold R. Kappos L. Arnold D. L. Bar-Or A. Giovannoni G. Selmaj K. . (2012). Placebo-controlled phase 3 study of oral BG-12 for relapsing multiple sclerosis. N. Engl. J. Med. 367, 1098–1107. 10.1056/NEJMoa1114287 22992073

[B8] Guazzi M. Arena R. Halle M. Piepoli M. F. Myers J. Lavie C. J. . (2018). 2016 focused update: clinical recommendations for cardiopulmonary exercise testing data assessment in specific patient populations. Eur. Heart J. 39, 1144–1161. 10.1093/eurheartj/ehw180 27141094

[B9] Harding A. E. (1981). Friedreich's ataxia: a clinical and genetic stud of 90 families with analysis of early diagnostic criteria and intrafamilial clustering of clinical features. Brain 104, 589–620. 10.1093/brain/104.3.589 7272714

[B10] Hayashi G. Cortopassi G. (2016). Lymphoblast oxidative stress genes as potential biomarkers of disease, severity, and drug effect in Friedreich's Ataxia. PLoS ONE 11, e0153574. 10.1371/journal.pone.0153574 27078885 PMC4831832

[B11] Hayashi G. Jasoliya M. Sahdeo S. Saccà F. Pane C. Filla A,. . (2017). Dimethyl fumarate mediates Nrf2-dependent mitochondrial biogenesis in mice and humans. Hum. Mol. Genet. 26, 2864–2873. 10.1093/hmg/ddx167 28460056 PMC6251607

[B12] Jasoliya M. Saccà F. Sahdeo S. Chedin F. Pane C. Brescia Morra V. . (2019). Dimethyl fumarate dosing in humans increases frataxin expression: a potential therapy for Friedreich ataxia. PLoS ONE 14, e0217776. 10.1371/journal.pone.0217776 31158268 PMC6546270

[B13] Kalinin S. Polak P. E. Lin S. X. Braun D. Guizzetti M. Zhang X. . (2013). Dimethyl fumarate regulates histone deacetylase expression in astrocytes. J. Neuroimmunol. 263, 13–19. 10.1016/j.jneuroim.2013.07.007 23916696

[B14] Libri V. Yandim C. Athanasopoulos S. Loyse N. Natisvili T. Law P. P. . (2014). Epigenetic and neurological effects and safety of high-dose nicotinamide in patients with Friedreich's ataxia. Lancet 384, 504–513. 10.1016/S0140-6736(14)60382-2 24794816

[B15] Linker R. A. Lee D.-H. Ryan S. van Dam A. M. Conrad R. Bista P. . (2011). Fumaric acid esters exert neuroprotective effects in neuroinflammation via activation of the Nrf2 antioxidant pathway. Brain 134, 678–692. 10.1093/brain/awq386 21354971

[B16] Litjens N. H. R. Burggraaf J. van Strijen E. van Gulpen C. Mattie H. Schoemaker R. C. . (2004). Pharmacokinetics of oral fumarates in healthy subjects. Br. J. Clin. Pharmacol. 58, 429–432. 10.1111/j.1365-2125.2004.02145.x 15373936 PMC1884599

[B17] Lynch D. R. Chin M. P. Delatycki M. B. Subramony S. H. Corti M. Hoyle J. C. . (2021). Safety and efficacy of omaveloxolone in Friedreich ataxia (MOXIe Study). Ann. Neurol. 89, 212–225. 10.1002/ana.25934 33068037 PMC7894504

[B18] Lynch D. R. Perlman S. L. Meier T. (2010). A phase 3, double-blind, placebo-controlled trial of idebenone in Friedreich ataxia. Arch. Neurol. 67, 941–947. 10.1001/archneurol.2010.168 20697044

[B19] Mrowietz U. Szepietowski J. C. Loewe R. van de Kerkhof P. Lamarca R. Ocker W. G. . (2017). Efficacy and safety of LAS41008 (dimethyl fumarate) in adults with moderate-to-severe chronic plaque psoriasis:a randomized, double-blind, Fumaderm(^®^) - and placebo-controlled trial (BRIDGE). Br. J. Dermatol. 176, 615–623. 10.1111/bjd.14947 27515097

[B20] Nguyen T. Sherratt P. J. Pickett C. B. (2003). Regulatory mechanisms controlling gene expression mediated by the antioxidant response element. Annu. Rev. Pharmacol. Toxicol. 43, 233–260. 10.1146/annurev.pharmtox.43.100901.140229 12359864

[B21] Pane C. Costabile T. Salvati A. Aurisicchio D. L. Abate F. Liguori A. . (2018). Adult normative values for the PATA Rate Test. J. Neurol. 265, 1102–1105. 10.1007/s00415-018-8820-0 29511862

[B22] Paupe V. Dassa E. P. Goncalves S. Auchère F. Lönn M. Holmgren A. . (2009). Impaired nuclear Nrf2 translocation undermines the oxidative stress response in Friedreich ataxia. PLoS ONE 4, e4253. 10.1371/journal.pone.0004253 19158945 PMC2617762

[B23] Pritchard A. Burns P. Correia J. Jamieson P. Moxon P. Purvis J. . (2021). ARTP statement on cardiopulmonary exercise testing 2021. BMJ Open Respir Res. 8, e001121. 10.1136/bmjresp-2021-001121 34782330 PMC8593741

[B24] Reetz K. Dogan I. Hilgers R.-D. Giunti P. Parkinson M. H. Mariotti C. . (2021). Progression characteristics of the European Friedreich's Ataxia Consortium for Translational Studies (EFACTS): a 4-year cohort study. Lancet Neurol. 20, 362–372. 10.1016/S1474-4422(21)00027-2 33770527

[B25] Reetz K. Dogan I. Hilgers R. D. Giunti P. Mariotti C. Durr A. . (2016). Progression characteristics of the European Friedreich's Ataxia Consortium for Translational Studies (EFACTS): a 2 year cohort study. Lancet Neurol. 15, 1346–1354. Erratum in: Lancet Neurol. (2017) 16, 954. 10.1016/S1474-4422(16)30287-3 27839651

[B26] Rostami-Yazdi M. Clement B. Mrowietz U. (2010). Pharmacokinetics of anti-psoriatic fumaric acid esters in psoriasis patients. Arch. Dermatol. Res. 302, 531–538. 10.1007/s00403-010-1061-4 20574745

[B27] Saccà F. Puorro G. Antenora A. Marsili A. Denaro A. Piro R. . (2011). A combined nucleic acid and protein analysis in friedreich ataxia. PLoS ONE 6, e17627. 10.1371/journal.pone.0017627 21412413 PMC3055871

[B28] Saccà F. Puorro G. Marsili A. Antenora A. Pane C. Casali C. . (2016). Long-term effect of Epoetin alfa on clinical and biochemical markers in Friedreich Ataxia. Mov. Disord. 31, 734–741. 10.1002/mds.26552 26879839

[B29] Sahdeo S. Scott B. D. McMackin M. Z. Jasoliya M. Brown B. Wulff H. . (2014). Dyclonine rescues frataxin deficiency in animal models and buccal cells of patients with Friedreich's ataxia. Hum. Mol. Genet. 23, 6848–6862. 10.1093/hmg/ddu408 25113747 PMC4245046

